# Negative correlation between the Bern score and opening pressure in myelography positive spontaneous intracranial hypotension

**DOI:** 10.1002/brb3.3409

**Published:** 2024-02-05

**Authors:** Dan Zhang, Qiaowei Zhang, Feifang He, Xingyue Hu, Jin Wang

**Affiliations:** ^1^ Department of Neurology Sir Run Run Shaw Hospital, Zhejiang University, School of Medicine Hangzhou China; ^2^ Department of Radiology School of Medicine Sir Run Run Shaw Hospital Zhejiang University Hangzhou China; ^3^ Department of Pain Management Center for Intracranial Hypotension Management Sir Run Run Shaw Hospital Zhejiang University, School of Medicine Hangzhou China

**Keywords:** brain magnetic resonance imaging, cerebrospinal fluid leak, opening pressure, spontaneous intracranial hypotension

## Abstract

**Objective:**

The Bern score is based on brain magnetic resonance imaging (MRI) to predict the probability of cerebrospinal fluid (CSF) leak in spontaneous intracranial hypotension (SIH). The aim of this study is to investigate the association between lumbar puncture opening pressure (OP) and the Bern score.

**Methods:**

We retrospectively reviewed OP measurement records and neuroimaging of patients with SIH in our center. The Bern score and its components were measured based on contrast‐enhanced brain MRI. The associations between OP and the Bern score, as well as its components, were analyzed. Patients were divided as low‐pressure (LP) group (OP < 60 mmH_2_O) and not‐low‐pressure (NLP) group (OP ≥ 60 mmH_2_O). Differences in terms of the Bern score and its components were compared between the two groups.

**Results:**

Seventy‐one (mean age 40.4 ± 10.6 years) patients with myelography confirmed CSF leak were included in this study. The mean disease duration when performed brain MRI was 32 ± 29 days, with a mean Bern score of 5.1 ± 2.7 and a mean OP of 68.6 ± 60.3 mmH_2_O. There are statistically negative correlations between OP and the Bern score (*p* < .001), as well as suprasellar cistern (*p* < .01) and prepontine cistern (*p* < .01). The presence of venous engorgement (*p* < .01) and pachymeningeal enhancement (*p* < .001) were significantly associated with OP. The LP groups have higher Bern scores than the NLP group (5.9 ± 2.5 vs. 4.2 ± 2.6, *p* = .004).

**Conclusions:**

A higher Bern score is indicative of not only an increased likelihood of a CSF leak but also a greater probability of low OP in patients with SIH. For patients with a Bern score ≥5 and positive heavily T2‐weighted MR myelography findings, epidural blood patch is reasonable before invasive myelography.

## Graphical abstract

This study shows negative correlation between the Bern score and opening pressure in spontaneous intracranial hypotension. Our finding suggests that a higher Bern score is indicative of not only an increased likelihood of a CSF leak, but also a greater probability of low opening pressure. For patients with a Bern score ≥5, promptly CSF leak treatment based on brain MR and heavily T2‐weighted MR myelography is reasonable before invasive evaluations.

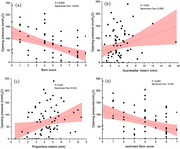

## 1 INTRODUCTION

Spontaneous intracranial hypotension (SIH) is caused by spinal cerebrospinal fluid (CSF) leak, with orthostatic headache being its most frequent symptom (Schievink, 2006). Delay or misdiagnosis of SIH is not uncommon (Callen et al., 2023; Dobrocky, Nicholson et al., 2022). In this setting, different tools are evolving to facilitate its diagnosis and detecting of CSF leak. The Bern score is a simple and easy‐to‐use probabilistic scoring system based on brain magnetic resonance imaging (MRI) to predict the probability of CSF leak in SIH (Dobrocky et al., 2019). The score system ranges from 0 to 9, where 0 indicates a low probability of spinal CSF leak and 9 indicates a high probability. In this study, we sought to investigate the association between lumbar puncture opening pressure (OP) and the Bern score, as well as with its components. The differences between patients with low OP and patients with normal or elevated OP in terms of the Bern score and its components were also compared.

## 2 METHODS

### 2.1 Study population

Between January 1, 2019 and December 31, 2021, 720 patients with a clinical suspicion of SIH underwent clinical work‐up at Sir Run Run Shaw Hospital, School of Medicine, Zhejiang University. The inclusion criteria of this study included: (1) patients met diagnostic criteria for SIH according to the third edition of the International Classification of Headache Disorders (ICHD‐3); (2) patients with adequate baseline brain contrast‐enhanced MRI performed prior to first ever epidural blood patch (EBP) or CSF leak surgery; (3) patients with lumbar puncture OP measurement record in our center. It should be noted that in our center lumbar puncture is performed for myelography, not just for measurement of OP; (4) CSF leak was confirmed by heavily T2‐weighted magnetic resonance myelography (MRM) and/or computed tomographic myelography (CTM). SIH caused by CSF‐venous fistula may demonstrate negative result in MRM or CTM, digital subtraction myelography (DSM) is required in these patients to localize CSF leak. Because our center has not yet conducted DSM, so CSF‐venous fistula patients were not included in this study. The exclusion criteria included: (1) secondary intracranial hypotension, for instance, post‐dural puncture headache; (2) symptoms of intracranial hypotension relieved upon admission; (3) unclear diagnosis of intracranial hypotension or with no proven CSF leak; (4) inadequate brain MRI (without contrast‐enhanced brain MRI, MRI with artifact resulting in the inability to evaluate the Bern score, or MRI acquired >2 weeks time interval between lumbar puncture); (5) age younger than 18 years.

Retrospectively, we reviewed medical records and neuroimaging of patients included. This study was approved by the Institutional Review Board Committee (Project ID 20210729‐075), which waived the requirement of written informed consent due to the retrospective study design.

### 2.2 Brain MRI

The brain MRI before EBP was performed on a 1.5‐ (UIH μMR560) or 3‐T scanner (GE signa HDxt). Routine MR imaging sequences included precontrast transverse T1‐weighted, traverse T2‐weighted, transverse fluid‐attenuated inversion recovery, and diffusion‐weighted MR images. Postcontrast transverse, sagittal, and coronary T1‐weighted images were acquired.

### 2.3 The Bern score

We applied the Bern score defined by Dobrocky, et al. (2019) originally published on JAMA Neurology 2019, which integrates six imaging findings in brain MRI: three major (two points each), engorgement of venous sinus, pachymeningeal enhancement, and effacement of the suprasellar cistern (≤4.0 mm) and three minor (one point each), subdural fluid collection, effacement of the prepontine cistern (≤5.0 mm), and mamillopontine distance (≤6.5 mm) (Dobrocky, et al., 2019). As defined by Dobrocky et al. (2019), patients with a Bern score of ≤2, 3–4, or ≥5 have a low, intermediate, or high probability of spinal CSF leak, respectively. Some scholars proposed a restricted Bern score in which the presence or absence of pachymeningeal enhancement was excluded from the scoring criteria (Callen et al., 2023). We also investigate the association between OP and restricted Bern score in this study. In patients for whom multiple brain MRIs were available in our center, the one with shortest time interval between lumbar puncture was adopted.

### 2.4 Heavily T2‐weighted MRM

Heavily T2‐weighted myelography was performed with a phase‐array spine coil on a 1.5‐T superconducting system (UIH μMR560). Axial MRMs were acquired at three levels (cervicothoracic, thoracic, and thoracolumbar), which overlapped at the margins, with the following parameters: repetition time 6000 ms, echo time 418 ms, matrix size 320 × 192, filed of view 224 × 224 mm, and slice thickness 6 mm. Post‐acquisition multiplanar reformation was performed to obtain axial (slice thickness 2 mm, gap 2 mm) and coronal images (slice thickness 2 mm, gap 2 mm).

### 2.5 Computed tomographic myelography (CTM)

A lumbar puncture through the L 3–4 or L 4–5 spinal interspace was performed in left lateral decubitus position with a 25‐ga spinal needle. After OP measurement, diluted nonionic iodinated contrast material (10 mL Iohexol, GE medical system, prediluted with 10 mL sterile 0.9% saline) was slowly injected around 10 min. The patient was then placed in the supine, right, prone, and left position in sequences, changing positions every 5 min, with a pillow under the buttocks. The patient was transferred at the same time to prepare for a CT scan. Helical CT (Siemens SOMATOM Definition Flash) scan from upper cervical to lower lumbar spine was obtained about half an hour after contrast injection. The images were reconstructed into axial planes (slice thickness 1.5 mm), sagittal planes (slice thickness 1.5 mm), and coronal planes (slice thickness 1 mm).

### 2.6 Image analysis

**FIGURE 1:**
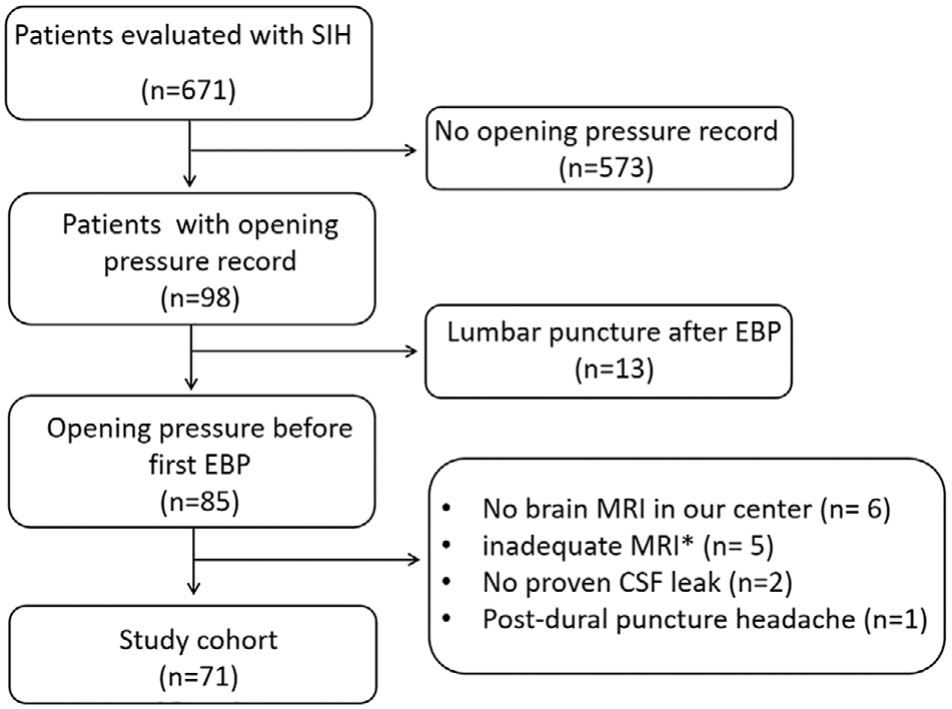
Study flowchart. Cerebrospinal fluid (CSF) cerebrospinal fluid, magnetic resonance imaging (MRI) magnetic resonance imaging, and spontaneous intracranial hypotension (SIH) spontaneous intracranial hypotension. * inadequate brain MRI stands for brain MRI without contrast‐enhanced, MRI with artifacts resulting in the inability to evaluate the Bern score, or MRI acquired >2 weeks time interval between lumbar puncture.

All radiological imaging was reviewed by at least one board‐certified neuroradiologist and one board‐certified neurologist. When a disagreement between readers in any signs was identified, the finding was reviewed together to reach consensus (flowchart see Figure 1).

### 2.7 Statistical analysis

Descriptive analysis used frequencies and percentages for categorical variables and mean ± SD for continuous variables. Univariable regression models were used to evaluate the correlation between OP and the Bern score or its components. Spearman correlation analysis was used for continuous variables, whereas Kendall correlation analysis was used for categorical variables. According to the OP, we also divided patients as low‐pressure (LP) group (<60 mmH2O) and not‐low‐pressure (NLP) group (≥60 mmH2O). Nonparametric analyses and Fisher's exact tests were used to compare categorical and continuous variables, respectively, between the two groups.

## 3 RESULTS

The final study cohort included 71 SIH patients with myelography confirmed CSF leak (LP: 37 patients and NLP: 34 patients; Table 1). Mean age was 40.4 ± 10.6 years (range 25–69 years, LP: 38.4 ± 9.8 years, and NLP: 42.75 ± 11.1 years). The study included 70.4% (50/71) women (LP: 75.7% [28/37] and NLP 64.7% [22/34]).

**TABLE 1:** Patients with opening pressure record and myelography confirmed CSF leak.

	Total (*n* = 71)	Low‐pressure (LP, *n* = 37)	Not‐low‐pressure (NLP, *n* = 34)	*p*‐Value
Age	40.4 ± 10.6	38.4 ± 9.8	42.5 ± 11.1	.075
Gender				.436
Female	50 (70.4%)	28 (75.7%)	22 (64.7%)	
Male	21 (29.6%)	9 (24.3%)	12 (35.3%)	
Opening pressure	68.6 ± 60.3	20.2 ± 19.9	121.2 ± 42.3	
Duration of symptoms when performed brain MRI (mean ± SD, days)	32 ± 29	30 ± 29	33 ± 30	.890
Bern score	5.1 ± 2.7	5.9 ± 2.5	4.2 ± 2.6	.004
Bern score grade				.025
Low (0–2)	15 (21.1%)	5 (13.5%)	10 (29.4%)	
Intermediate (3–4)	15 (21.1%)	5 (13.5%)	10 (29.4%)	
High (5–9)	41(57.7%)	27 (73.0%)	14 (41.2%)	
Restricted Bern score	4.1 ± 2.1	4.6 ± 2.0	3.5 ± 2.0	.025
Venous engorgement	46 (64.8%)	27 (73.0%)	19 (55.9%)	.146
Pachymeningeal enhancement	36 (50.7%)	25 (67.6%)	11 (32.4%)	.004
Subdural collection	13 (18.3%)	6 (16.2%)	7 (20.6%)	.762
Suprasellar cistern (mean ± SD)	3.9 ± 2.7	3.3 ± 2.2	4.6 ± 3.1	.044
≤4 mm	42 (59.2%)	25 (67.6%)	17 (50.0%)	.154
Prepontine cistern (mean ± SD)	4.3 ± 1.6	3.9 ± 1.5	4.6 ± 1.6	.063
≤5 mm	51 (71.8%)	30 (81.1%)	21 (61.8%)	.112
Mamillopontine distance (mean ± SD)	5.8 ± 1.6	5.9 ± 1.6	5.7 ± 1.7	.809
≤ 6.5 mm	49 (69.0%)	28 (75.7%)	21 (61.8%)	.304

*Note*: All the brain MRIs were performed before first‐ever epidural blood patch or CSF leak surgery. The time intervals between brain MRI scanning and lumbar puncture were within 2 weeks. The Bern score ranges from 0 to 9, with 0 indicating very low and 9 very high probability of spinal CSF leak. Data are number (%) or mean (SD). Nonparametric analyses were used for continuous variables. Fisher's exact tests were used for categorical variables.

Abbreviations: CSF, cerebrospinal fluid; MRI, magnetic resonance imaging; SD, standard deviation.

**FIGURE 2:**
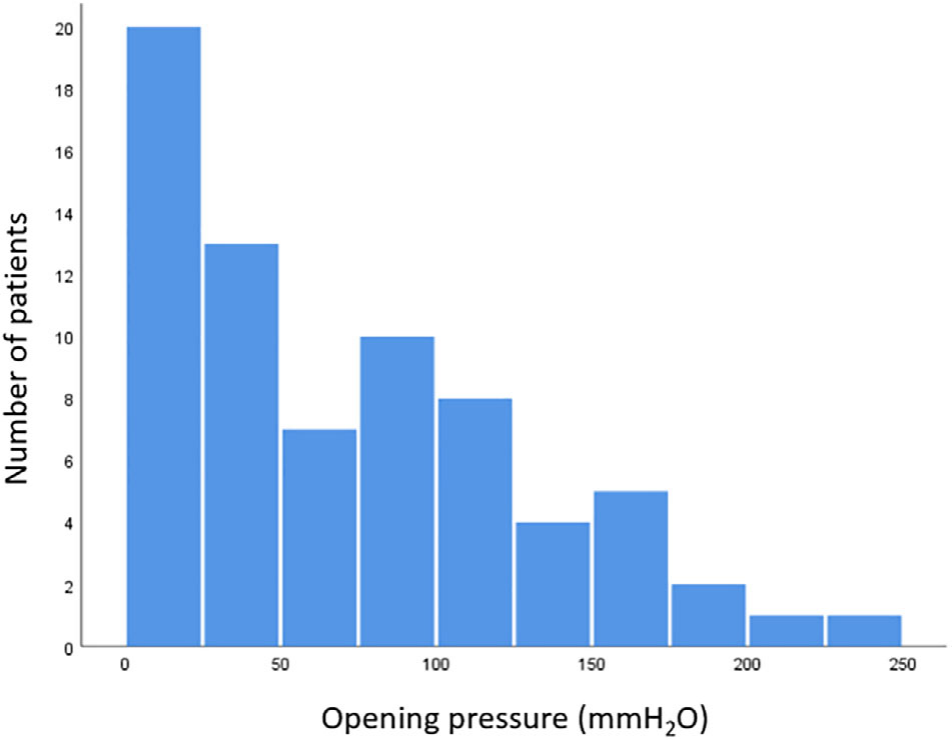
Histogram showing the distribution of opening pressures in individuals in our cohort.

**FIGURE 3:**
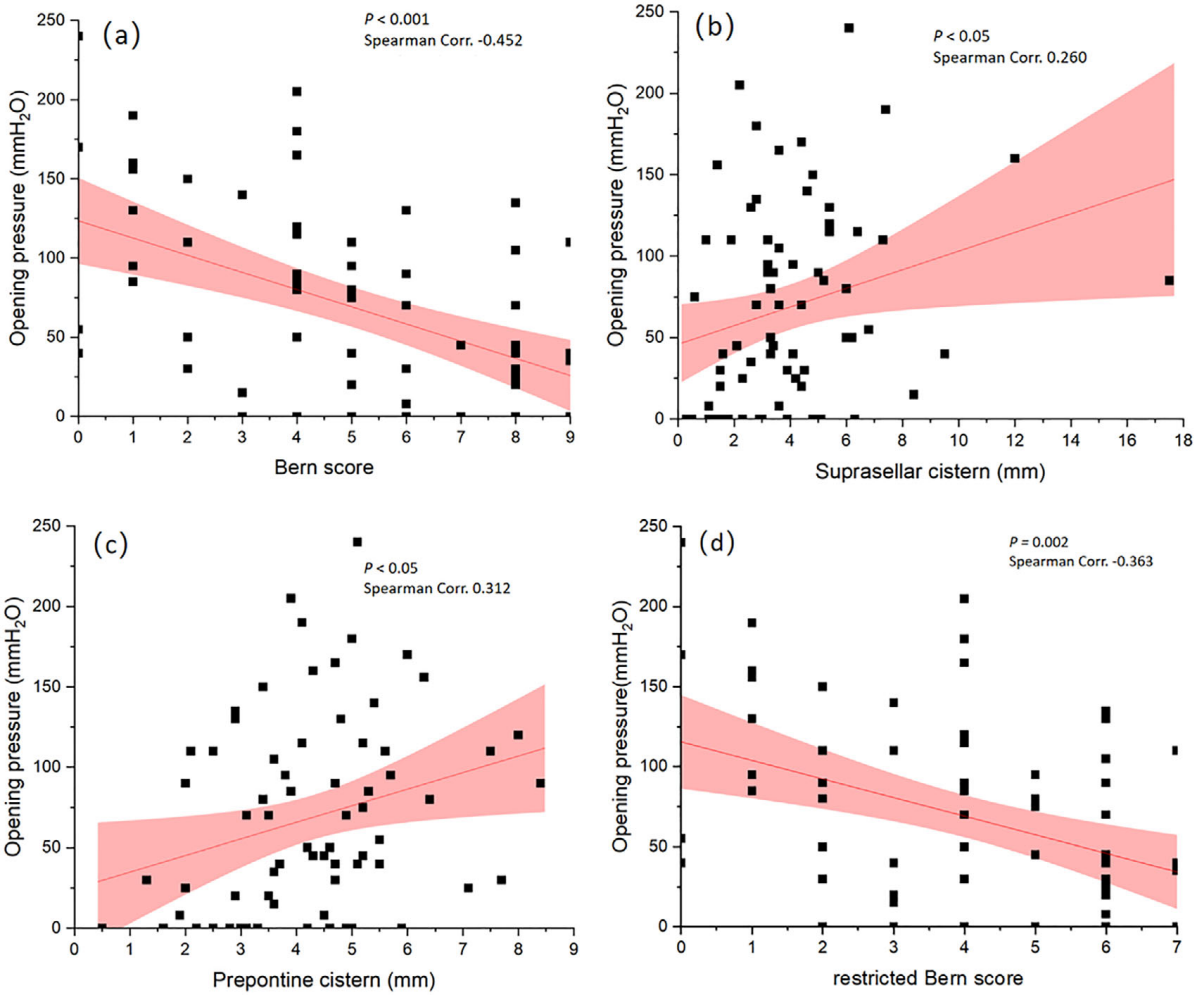
Correlation between opening pressure and the Bern score (a), as well as two continuous variables (b and c) of its six components. Correlation between opening pressure and the restricted Bern score (d).

The mean duration of symptoms when performed MRI was 32 ± 29 days, with a mean Bern score of 5.1 ± 2.7, and a mean OP of 68.6 ± 60.3 mmH_2_O. The distribution of OP in our cohort is shown in Figure 2. There are statistically negative correlations between OP and the Bern score (*p* < .001), as well as suprasellar cistern (*p* < .01) and prepontine cistern (*p* < .01) (Table 2 and Figure 3). The presence of venous engorgement (*p* < .01) and pachymeningeal enhancement (*p* < .001) were significantly associated with OP (Table 2). A negative correlation was also found between OP and the restricted Bern score (*p =* .002) (Table 2 and Figure 3).

**TABLE 2:** Results of correlation analysis investigating the relationship of individual components of the Bern score to opening pressure.

	Opening pressure	
	*p*‐Value	Correlation coefficient
Bern score	.000	−.452^a^
Venous engorgement	.007	−.271^b^
Pachymeningeal enhancement	.000	−.392^b^
Subdural collection	.846	.020^b^
Suprasellar cistern	.005	.329^a^
Prepontine cistern	.008	.312^a^
Mamillopontine distance	.702	.046^a^
Restricted Bern score	.002	−.363^a^

^a^ Spearman correlation analysis.

^b^ Kendall correlation analysis.

**FIGURE 4:**
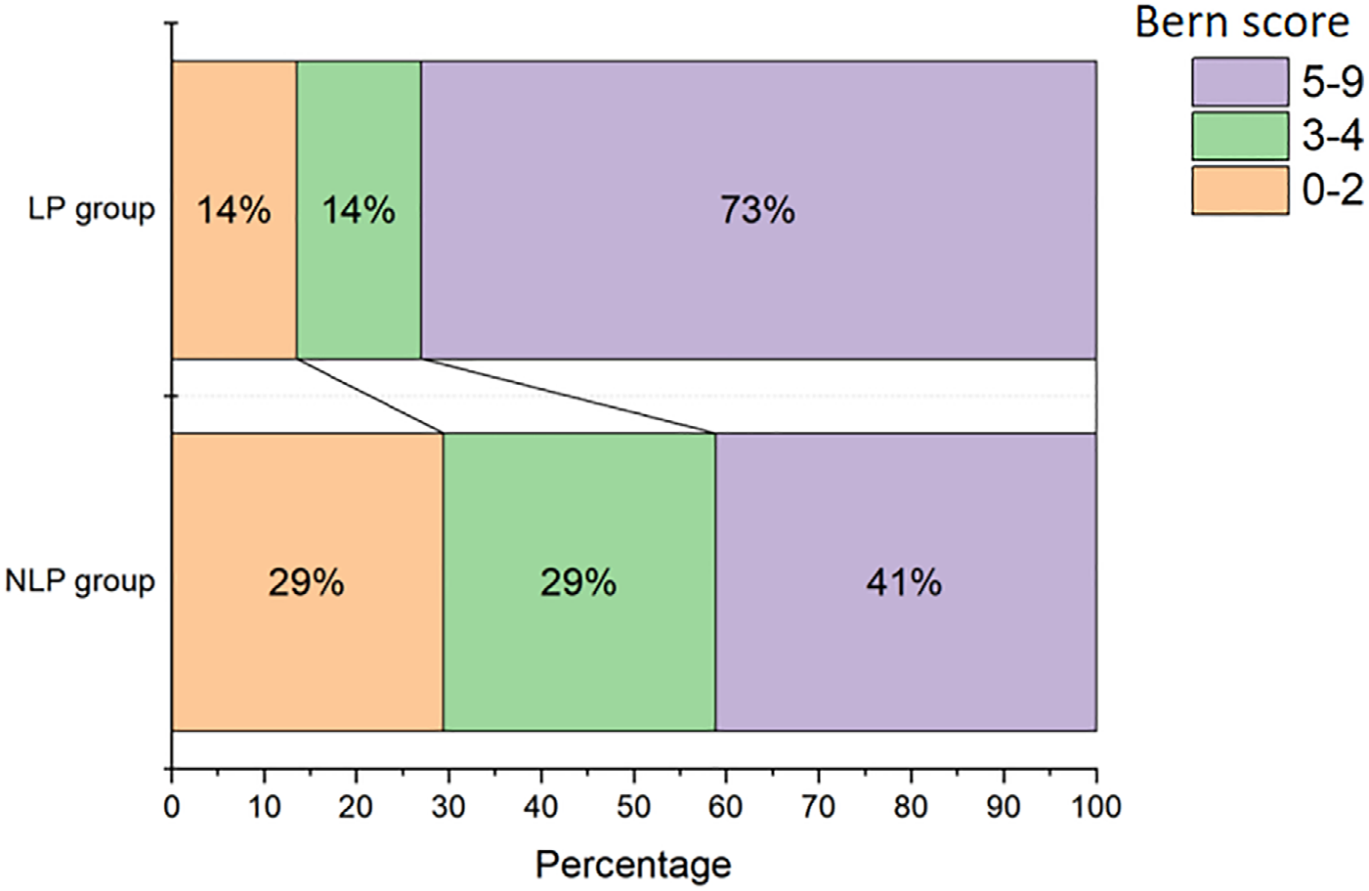
The distributions of Bern score in low‐pressure (LP) group and not‐low‐pressure (NLP) group (*p* = .025). The Bern score ranges from 0 to 9. Patients with a score of 0–2 have a low, 3–4 an intermediate, and 5–9, a high probability of spinal cerebrospinal fluid (CSF) leak.

The Bern scores are larger in the LP group than those in the NLP group (5.9 ± 2.5 vs. 4.2 ± 2.6, *p* = .004). It is similar for restricted Bern score (LP 4.6 ± 2.0 vs. NLP 3.5 ± 2.0, *p* = .025) (Table 1). Correspondingly, there are also differences in the Bern score distributions between the two groups (*p* = .025) (Figure 4). In terms of different components of the Bern score, the percentage of patients with pachymeningeal enhancement (67.6% vs. 32.4%, *p* = .004) was higher in the LP group (Table 1). The height of suprasellar cistern (3.3 ± 2.2 mm vs. 4.6 ± 3.1 mm, *p* = .044) was shorter in the LP group (Table 1).

## 4 DISCUSSION

In this retrospective study, we found a statistically negative correlation between the Bern score and OP in patients with myelography confirmed SIH. Patients with low OP overall have larger Bern scores than patients with normal or elevated pressure.

According to ICHD‐3, either an OP < 60 mmH_2_O on lumbar puncture or typical neuroimaging findings of intracranial hypotension (either direct on spine imaging or indirect on brain imaging) are required for a diagnosis of SIH (Callen et al., 2023; ICHD‐3, 2018). However, contrary to what its name might suggest, about half of the patients with CT myelography confirmed CSF leak have normal or even elevated lumbar puncture OP, as shown in previous studies (Kranz, Tanpitukpongse et al., 2016; Yao & Hu, 2017). Therefore, OP < 60 mmH_2_O is not a necessary condition for diagnosing SIH. Of importance in turn, OP should not be used to exclude patients from further diagnostic process when there is clinical suspicion for SIH (Callen et al., 2023).

Based on different etiology, morphology, and distance from the midline, CSF leak was classified into four types by scholars (Schievink et al., 2016; Farb et al., 2019). Type 1 is caused by degenerative disc disease creating a mechanical tear in the ventral dura. Type 2 leaks due to a more lateral tear near neural foramina, usually combined with meningeal diverticula. Type 3 is caused by a direct CSF‐venous fistulas. However, type 4 results from a distal nerve root sleeve leak into the adjacent facial planes (Farb et al., 2019).

Neuroimaging is crucial in diagnosing, classification, and monitoring SIH (Schievink, 2016; Dobrocky et al., 2022), especially as low OP is an unreliable marker. Various imaging tools are used to diagnose and localize CSF leak. Currently, dynamic myelography is required to localize a CSF leak precisely, but this is invasive, time‐consuming, costly, and ionizing radiative. Besides, it may also expose the patient to complications, especially for those patients who already have subdural hematomas. Besides invasive myelography, heavily T2‐weighted MRM is a noninvasive, non‐contrasted, and time‐saving method for assessing the location of CSF leak. Scholars have compared these two different ways of myelography, which indicates that heavily T2‐weighted MRM was accurate in localizing CSF leaks and could be an alternative to invasive myelography before targeted EBPes (Wang et al., 2009; Tay et al., 2021). But MRM or CTM cannot detect or localize a CSF‐venous fistula (type 3 leak), whereas DSM is required (Schievink et al., 2016).

In clinical practice, brain MRI is usually done first in patients presenting with new onset headaches. To standardize the diagnostic pathways of patients with SIH, Dobrocky et al. (2019) proposed this 3‐tier predictive scoring system (the Bern score), which is based on the six most relevant brain MRI findings. Patients with a Bern score of ≤2, 3–4, or ≥5 have a low, intermediate, or high probability of spinal CSF leak, respectively (Dobrocky et al., 2019). Although in the cohort developing the Bern score, SIH patients caused by CSF‐venous fistula were not included (Dobrocky et al., 2019), recent studies indicate that the Bern score could also predict CSF‐venous fistula (Callen et al., 2023; Kim et al., 2021). A recent study shows that the Bern score could also serve as a reliable quantitative tool to monitor treatment success in SIH patients (Dobrocky et al., 2022).

Suggested by Dobrocky et al. (2019), for patients with a Bern score ≥5, considering the high probability of positive findings in myelography, EBP should be considered promptly without performing myelography. In our study, the mean Bern score is above 5. Besides, the Bern score of LP group was larger than 5, whereas that of the NLP group is smaller than 5. Based on our clinical practice as well as suggested by literature, for patients with OP < 60 mmH_2_O, the likelihood of contrast injection failure (extrathecal contrast injection) is high due to changes in spinal membrane elasticity during myelography (Beck et al., 2017; Xu et al., 2018). Therefore, our study also supports the recommendation that for patients with a Bern score ≥5 and positive heavily T2‐weighted MRM findings, targeted EBP is reasonable before an invasive myelography. Although our study did not included CSF‐venous fistula patients, considering this type of CSF leak is usually negative in MRM and refractory to EBP, this recommendation also has its rationality.

Callen et al. (2023) illustrated the average Bern score in individuals with a CSF leak was significantly higher than in those without. In our study, we reported a statistically negative correlation between the Bern score and OP in patients with invasive myelography confirmed SIH. The Bern score contains two main pathophysiologic variables in SIH: (1) those relating to increased intracranial vascular volume replacing loss of CSF, including venous engorgement, pachymeningeal enhancement, and subdural fluid collection and (2) those structural changes reflecting brain sagging due to reduced buoyancy, including reduced prepontine and mamillopontine distance (Chen et al., 2022). In our study, we find venous engorgement; pachymeningeal enhancement, suprasellar distance, and prepontine distance are associated with OP. When comparing patients with low OP and not‐low OP, pachymeningeal enhancement and suprasellar cistern were different between the two groups. Previous studies illustrate that there is no difference between LP and NLP SIH in terms of symptoms, neuroimaging, and response to treatment, except that the NLP group have longer disease duration (Chen et al., 2022; Mokri, 1999). Shun‐Jiun Wang et al. found that cerebral venous dilation‐related brain MRI findings generally developed earlier than brain deformity‐related brain MRI findings (Chen et al., 2022). Therefore, the difference between low OP and not‐low OP patients may lie primarily in different dominant compensation mechanisms. Patients with low OP may have more obvious venous dilation due to the low intracranial pressure, which consists of the Monro–Kellie postulate. While in patients with normal or elevated pressure, the key problem may lie in decreased buoyancy due to low CSF volume. Besides intracranial factors, dilation of the intraspinal venous structures, spinal elastance, and body habitus, may also affect OP (Beck et al., 2017; Callen et al., 2023; Kranz et al., 2016). OP may be determined in a more complex manner than has been generally understood. Future work is needed to address these questions.

Among the five typical neuroimaging findings in the brain MR enhanced with contrast (known as SEEPS), the occurrence of pachymeningeal enhancement is highest, with an incidence of approximately 80% (Dobrocky et al., 2019; Kranz, et al., 2016). Therefore, pachymeningeal enhancement is a neuroimaging finding of important diagnostic value. Although it also needs to be differentiated from other diseases, such as hypertrophic pachymeningitis, that cause dural enhancement. The research of Shun‐Jiun Wang et al. shows the emergence of brain MRI findings of SIH depends on disease duration (Chen et al., 2022). The incidence of diffuse pachymeningeal enhancement grows with time, if CSF leak persists, with an incidence of about 50% in the first week and 82.5% after 1 month (Chen et al., 2022). However, most patients with headache usually received MRI without contrast, especially when initially SIH was not a suspicion, and some patients with renal insufficiency may have contraindication for gadolinium use, in these scenarios, the restricted Bern score would has practical utility. Callen et al. (2023) found restricted Bern score continued to be strongly associated with the presence of a CSF leak. Our study also found a negative correlation between OP and restricted Bern score. A difference in restricted Bern score was also found between LP and normal pressure SIH patients. Despite all this, given that pachymeningeal enhancement is still the most diagnostic imaging feature, the application of restricted Bern score and its proper cutoff still needs to be validated in more cohorts.

Our study has several limitations. First, referral bias may exist as our center is the largest center for SIH management in China mainland. Many of our patients were transferred from other provinces and had unsuccessful initial treatment at local hospitals. Second, lumbar puncture was not routinely performed in all the patients with a suspicion of SIH in our center. For patients with typical symptoms, typical brain MRI and heavily T2‐weighted MRM, or patients with severe complications, such as subdural hematoma or cerebral venous thrombosis, conventional myelography was not performed. Therefore, according to our research purpose, inclusion and exclusion criteria, some clinical characteristics of patients in our cohort may not represent the overall characteristics of SIH patients. For example, the mean Bern score in our cohort does not represent the overall mean Bern score of patients with SIH. Third, because our center has not yet conducted DSM, conventional invasive myelography was used to confirm a CSF leak. But this method cannot diagnose patients with CSF‐venous fistula (Dobrocky et al., 2022). Fourth, our study is retrospective and monocentric with modest sample size.

Although previous study showed that a higher Bern score is not necessarily indicative of more severe cases of SIH (Dobrocky et al., 2019). The onset‐neuroimaging interval is also suggested to be considered when using brain MRI finding score system for diagnostic purposes (Chen et al., 2022). But all in all, our study supports that a Bern score ≥5 is of important diagnostic and guiding management value in patients with SIH in Chinese population.

## 5 CONCLUSION

The Bern score is also a simple and easy‐to‐use diagnostic tool to predict the probability of CSF leak in Chinese population. A higher Bern score is indicative of not only an increased likelihood of a CSF leak but also a greater probability of low OP. For patients with a Bern score ≥5, promptly EBP based on brain MRI and heavily T2‐weighted MR myelography is reasonable before invasive evaluations.

## AUTHOR CONTRIBUTIONS

**Dan Zhang**: Conceptualization; methodology; data curation; investigation; formal analysis; visualization; writing—original draft; writing—review and editing; funding acquisition. **Qiaowei Zhang**: Methodology; software; investigation; formal analysis; writing—review and editing; data curation. **Feifang He**: Methodology; data curation; investigation; writing—review and editing; funding acquisition; formal analysis. **Xingyue Hu**: Methodology; project administration; writing—review and editing. **Jin Wang**: Writing—original draft; writing—review and editing; conceptualization; methodology; supervision; project administration; investigation; data curation; formal analysis.

## CONFLICT OF INTEREST STATEMENT

The authors declared they have no conflicts of interest.

## FUNDING INFORMATION

Medical and Health Project of Health Commission of Zhejiang Province, Grant No.: 2022KY846; Clinical Research Fund of Zhejiang Medical Association, Grant No.: 2021ZYC‐A06; and Zhejiang Province Public Welfare Research Projects, Grant No.: LGF20H090014

### PEER REVIEW

The peer review history for this article is available at https://publons.com/publon/10.1002/brb3.3409.

## Data Availability

Anonymized data not published in this article will be made available by request from any qualified investigator or appropriate collaborative researchers.

## References

[brb33409-bib-0001] Beck, J. , Fung, C. , Ulrich, C. T. , Fiechter, M. , Fichtner, J. , Mattle, H. P. , Mono, M. L. , Meier, N. , Mordasini, P. , Z'Graggen, W. J. , Gralla, J. , & Raabe, A. (2017). Cerebrospinal fluid outflow resistance as a diagnostic marker of spontaneous cerebrospinal fluid leakage. Journal of Neurosurgery Spine, 27(2), 227–234. 10.3171/2017.1.SPINE16548 28574328

[brb33409-bib-0002] Callen, A. L. , Pattee, J. , Thaker, A. A. , Timpone, V. M. , Zander, D. A. , Turner, R. , Birlea, M. , Wilhour, D. , O'Brien, C. , Evan, J. , Grassia, F. , & Carroll, I. R. (2023). Relationship of Bern score, spinal elastance, and opening pressure in patients with spontaneous intracranial hypotension. Neurology, 100(22), e2237–e2246. 10.1212/WNL.0000000000207267 37015821 PMC10259284

[brb33409-bib-0003] Chen, S. T. , Wu, J. W. , Wang, Y. F. , Lirng, J. F. , Hseu, S. S. , & Wang, S. J. (2022). The time sequence of brain MRI findings in spontaneous intracranial hypotension. Cephalalgia, 42(1), 12–19. 10.1177/03331024211044424 34579563

[brb33409-bib-0004] Dobrocky, T. , Grunder, L. , Breiging, P. S. , Branca, M. , Limacher, A. , Mosimann, P. J. , Mordasini, P. , Zibold, F. , Haeni, L. , Jesse, C. M. , Fung, C. , Raabe, A. , Ulrich, C. T. , Gralla, J. , Beck, J. , & Piechowiak, E. I. (2019). Assessing spinal cerebrospinal fluid leaks in spontaneous intracranial hypotension with a scoring system based on brain magnetic resonance imaging findings. JAMA Neurology, 76(5), 580–587. 10.1001/jamaneurol.2018.4921 30776059 PMC6515981

[brb33409-bib-0005] Dobrocky, T. , Nicholson, P. , Hani, L. , Mordasini, P. , Krings, T. , Brinjikji, W. , Cutsforth‐Gregory, J. K. , Schar, R. , Schankin, C. , Gralla, J. , Pereira, V. M. , Raabe, A. , Farb, R. , Beck, J. , & Piechowiak, E. I. (2022). Spontaneous intracranial hypotension: Searching for the CSF leak. Lancet Neurology, 21(4), 369–380. 10.1016/S1474-4422(21)00423-3 35227413

[brb33409-bib-0006] Dobrocky, T. , Hani, L. , Rohner, R. , Branca, M. , Mordasini, P. , Pilgram‐Pastor, S. , Kaesmacher, J. , Cianfoni, A. , Schar, R. T. , Gralla, J. , Raabe, A. , Ulrich, C. , Beck, J. , & Piechowiak, E. I. (2022). Brain spontaneous intracranial hypotension score for treatment monitoring after surgical closure of the underlying spinal dural leak. Clinical Neuroradiology, 32(1), 231–238. 10.1007/s00062-021-01124-z 35028683

[brb33409-bib-0007] Farb, R. I. , Nicholson, P. J. , Peng, P. W. , Massicotte, E. M. , Lay, C. , Krings, T. , & ter Brugge , K. G. (2019). Spontaneous intracranial hypotension: A systematic imaging approach for CSF leak localization and management based on MRI and digital subtraction myelography. AJNR: American Journal of Neuroradiology, 40(4), 745–753. 10.3174/ajnr.A6016 30923083 PMC7048504

[brb33409-bib-0008] Headache Classification Committee of the International Headache Society (IHS) . (2018). The international classification of headache disorders, 3rd edition. Cephalalgia, 38(1), 1–211. 10.1177/0333102417738202 29368949

[brb33409-bib-0009] Kim, D. K. , Carr, C. M. , Benson, J. C. , Diehn, F. E. , Lehman, V. T. , Liebo, G. B. , Morris, J. M. , Morris, P. P. , Verdoorn, J. T. , Cutsforth‐Gregory, J. K. , Atkinson, J. L. D. , & Brinjikji , W. (2021). Diagnostic yield of lateral decubitus digital subtraction myelogram stratified by brain MRI findings. Neurology, 96(9), e1312–e1318. 10.1212/WNL.0000000000011522 33472917

[brb33409-bib-0010] Kranz, P. G. , Tanpitukpongse, T. P. , Choudhuryn, K. R. , Amrhein, T. J. , & Gray, L. (2016). How common is normal cerebrospinal fluid pressure in spontaneous intracranial hypotension? Cephalalgia, 36(13), 1209–1217. 10.1177/0333102415623071 26682575

[brb33409-bib-0011] Kranz, P. G. , Luetmer, P. H. , Diehn, F. E. , Amrhein, T. J. , Tanpitukpongse, T. P. , & Gray , L. (2016). Myelographic techniques for the detection of spinal CSF leaks in spontaneous intracranial hypotension. AJR: American Journal of Roentgenology, 206(1), 8–19. 10.2214/AJR.15.14884 26700332

[brb33409-bib-0012] Mokri, B. (1999). Spontaneous cerebrospinal fluid leaks: From intracranial hypotension to cerebrospinal fluid hypovolemia—Evolution of a concept. Mayo Clinic Proceedings, 74(11), 1113–1123. 10.4065/74.11.1113 10560599

[brb33409-bib-0013] Schievink, W. I. (2006). Spontaneous spinal cerebrospinal fluid leaks and intracranial hypotension. JAMA, 295(19), 2286–2296. 10.1001/jama.295.19.2286 16705110

[brb33409-bib-0019] Schievink, W. I. , Maya, M. M. , Jean‐Pierre, S. , Nuño, M. , Prasad, R. S. , & Moser, F. G. (2016). A classification system of spontaneous spinal CSF leaks. Neurology, 87(7), 673–679. 10.1212/WNL.0000000000002986 27440149

[brb33409-bib-0020] Tay, A. S. , Maya, M. , Moser, F. G. , Nuno, M. , & Schievink, W. I. (2021). Computed tomography vs heavily T2‐weighted Magnetic resonance myelography for the initial evaluation of patients with spontaneous intracranial hypotension. JAMA Neurol, 78(10), 1275–1276. 10.1001/jamaneurol.2021.2868 34459855 PMC8406204

[brb33409-bib-0015] Wang, Y. F. , Lirng, J. F. , Fuh, J. L. , Hseu, S. S. , & Wang, S. J. (2009). Heavily T2‐weighted MR myelography vs CT myelography in spontaneous intracranial hypotension. Neurology, 73(22), 1892–1898. 10.1212/WNL.0b013e3181c3fd99 19949036

[brb33409-bib-0016] Xu, J. Q. , Wang, J. , & Gong, X. Y. (2018). Low tension of the dural sac as a cause of unsuccessful myelography in spontaneous intracranial hypotension: Evidence from computed tomographic‐guided myelography. Neurology India, 66(2), 518–520. 10.4103/0028-3886.227307 29547178

[brb33409-bib-0017] Yao, L. L. , & Hu, X. Y. (2017). Factors affecting cerebrospinal fluid opening pressure in patients with spontaneous intracranial hypotension. Journal of Zhejiang University Science B, 18(7), 577–585. 10.1631/jzus.B1600343 28681582 PMC5498838

